# Learning from history: the need for a synthetic approach to human cognition

**DOI:** 10.3389/fpsyg.2015.01435

**Published:** 2015-09-24

**Authors:** Bernhard Hommel, Lorenza S. Colzato

**Affiliations:** Cognitive Psychology Unit, Leiden Institute for Brain and Cognition, Leiden UniversityLeiden, Netherlands

**Keywords:** theory, research, attention, analytical modeling, synthetic

History does repeat itself if it comes to how cognitive science develops new research lines. Consider attention, one of the first experimentally researched psychological phenomenon. While William James still thought that “everyone knows what attention is,” it took us more than 100 years to learn that attention is actually not one coherent thing but falls into very different subfunctions, which again are subserved by various processes. It seems only natural that a young, developing scientific discipline needs some time to learn how to deal with phenomena, translate them into useful scientific concepts, and investigate the underlying mechanisms. And yet, we apparently did not learn much from this quest, as witnessed by the very similar developments in research on phenomena with a younger history. Developments showing the same problematic tendencies that have troubled attention research and slowed down its progress for/by many years. In the following, we shall try to capture the typical way new research lines develop—the *analytical approach*, as we call it, by using attention as an example. Then we focus on three pitfalls in this process that seem particularly difficult to circumvent and present an alternative research strategy—the *synthetic approach*.

## How research lines in cognitive science develop

**Figure 1:**
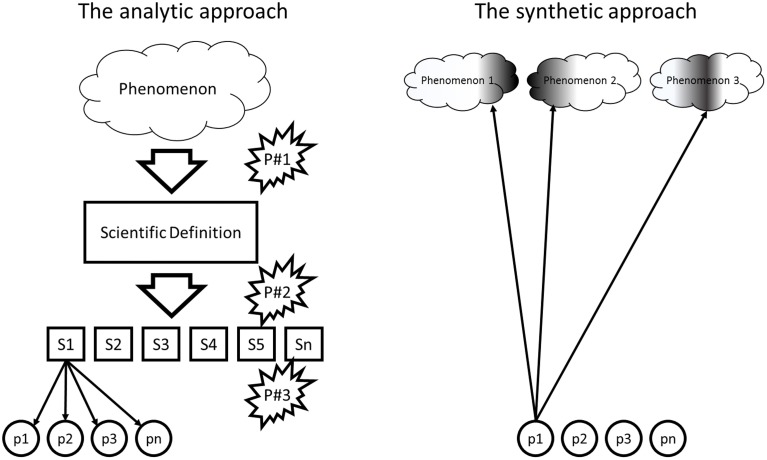
**The analytic and the synthetic approach to human cognition**. The dominating analytic approach involves translating a real-life phenomenon into a scientific definition, which later is split into definitions of subfunctions (S1, …. Sn) and mapped onto underlying processes (p1, … pn). The flash signs refer to the three pitfalls we discuss. The alternative synthetic approach consists in investigating which aspects of different phenomena are accounted for by a particular process. Its goal is not (necessarily) to account for entire phenomena but, rather, for many aspects of many phenomena by means of the same process.

While it is impossible to capture the rich history of attentional research in a short paragraph, the developments that were particularly relevant for our argument are sketched in Figure 1 (left panel). Psychological concepts commonly have a long history in layperson theorizing and philosophy. Both rely on introspection and thus tend to describe phenomena from a first-person perspective; e.g., William James refers to object-oriented attention by saying: “Attention to an object is what takes place whenever that object most completely occupies the mind” (James, 1899/2008, p. 63). As such personal-level descriptions are restricted to conscious experience, they are not useful for theorizing about the underlying processes and for generating hypotheses to guide empirical investigation. Accordingly, they need to be translated into, and defined by means of a more objective systems-level (or “sub-personal”: Dennett, 1969, following Ryle and Wittgenstein) language—attention becomes an “attentional system” or “attentional network.”

Attention research took some time to find out that there is not “the attention” but, rather, different subfunctions that often differ in their operational logic, functional characteristics, and the neural resources they rely on—just think of spatially focusing, feature integration, and vigilance. This insight has led to considerable segregation: Researchers working on one subfunction hardly talk to researchers working on another, use different terminology, gather in different symposia at different conferences, and publish in different journals. Decades of research on these subfunctions has increased our insight into the underlying processes, so that for each subfunction a number of underlying processes can be defined (see lowermost level of the figure). We developed this scenario for attention but similar stories could be told for other phenomena: The concept of human will saw the same transition from experience-based personal-level descriptions to systems-level terminology—*will* became *executive control* (Goschke, 2003). First understood as a single coherent system, recent research has motivated the distinction between different subfunctions that rely on different processes (Miyake et al., 2000).

## Three pitfalls

Each step in our sketch had its reasons and was probably inevitable for a young scientific discipline. And yet, there is little evidence that younger research lines have learned much from the less fortunate choices that have been made on the way. We shall focus on three pitfalls related to the three transitions from one level to the next, as indicated in Figure 1.

### Pitfall #1: searching for exhaustive definitions

Research on human cognition commonly takes its phenomena from everyday life: people attend to some things but not to others, which affects their experience and behavior, and the cognitive sciences need to explain why. The phenomena are originally described in everyday language, which is highly context-dependent and scientifically naïve. In everyday life, we do understand what people mean if they say “I couldn't attend class” and “it has come to my attention,” and we do not find it strange to use the same word to refer to acts of courtesy and a position assumed in military formation. While using the same word in all these situations may imply functional commonalities, there is no a priori reason why it should. Hence, there is no reason to even try capturing all these different meanings by one scientific definition and, as the example of attention shows, the attempt is likely to fail. This may seem obvious, but not only has attentional research taken quite some time to find that out, younger research lines seem to run into the exact same trap. For instance, creativity researchers still aim to develop tests assessing and models covering “the creativity”—endeavors that we consider likely to fail.

### Pitfall #2: identifying processes with subfunctions

Cognitive researchers find out how phenomena/functions work by identifying the underlying processes. Unfortunately, however, this is often accompanied by assuming a one-to-one mapping between the particular function and the respective set of processes—which then become a “feature-integration mechanism” or an “inhibition network.” This strategy has some unfortunate consequences.

Among other things, it implies that the underlying processes are specific to the particular subfunction and not shared by other subfunctions. But this is very unlikely and, as for instance a recent meta-review on the links between emotions and brain structures (Lindquist et al., 2012) shows, often inconsistent with the empirical evidence. The differentiation of phenomena into subfunctions is commonly based on the insight that the respective subfunctions are not the same, which does not mean that they have nothing in common. For instance, there is a widespread agreement that processing in the brain follows a winner-takes-all logic, which means that the activation or selection of one representation or state leads to the suppression of its competitors (Bogacz, 2007). This mechanism is likely to be an important ingredient of target selection, an important subfunction of attention, but also likely to be important for distractor suppression, feature integration, memory retrieval, or emotion regulation. Hence, being essential for one subfunction does not prevent a process from contributing to other subfunctions as well. The practical problem this raises is that researchers working on different subfunctions are likely to deal with the same processes without even knowing (given the segregation of researchers), which is why models in different subareas often have a rather similar architecture (e.g., almost all areas have dual-route processing models). A good example for this (understandable, given the research strategy) mutual ignorance is work on attention-switching and on theory of mind. While researchers from both communities have favored the temporo-parietal junction as one key system for their phenomena for some time already, efforts to appreciate and explain this commonality have been taken only recently (Geng and Vossel, 2013). Other recent efforts to overcome the limitations of earlier function-to-process mapping approaches can be found in the discussions on the mirror neuron system (Michael, 2011) and the role of the amygdala.

A concrete example for the problems that the identification of processes with their subfunctions can create comes from our own research. On the one hand, divergent thinking (a component of creativity) relies on striatal dopamine and can thus be improved by increasing the dopaminergic level. On the other hand, positive mood is associated with the striatal dopamine level. According to the analytical approach, this would render dopamine both a “creativity process” and a “mood process.” While that seems just a semantic issue, it does become relevant when trying to show (as we did) that increasing the dopamine level improves divergent thinking and reviewers require one to rule out that this is a mood effect. We are afraid that this problem is more general than one may think. If we consider that phenomena are derived from context-dependent everyday language, there is no reason to assume that concepts like attention, motivation, will, or emotion do not share any processes. But if they do, it makes little sense to pit one concept against another in empirical research. That is, it is logically impossible to decide whether some experimental effect was “attentional,” “motivational,” or “emotional” in nature, because this very nature is likely to overlap.

### Pitfall #3: specializing in phenomena rather than processes

As we have pointed out, researchers segregate according to the phenomena (or subfunctions) they are interested in. Segregation as such is difficult to avoid, as the sheer amount of work and the increasing methodological sophistication in all related areas makes it impossible to be on top of things in a whole discipline. What we find problematic, however, is the criterion for segregation. As we have pointed out, using phenomena or subfunctions as criterion creates various problems, including parallel attempts to reinvent wheels and building models of very similar structure without even knowing. Avoiding that requires a necessary counterweight, which we suggest can be provided by a synthetic approach.

## The synthetic approach

According to our analysis, circumventing the three discussed pitfalls requires not to: (a) search for exhaustive definitions and complete models; (b) identify processes with phenomena; and (c) specialize in phenomena. As the dominating analytic approach makes avoiding all that difficult, if not impossible, another approach is needed. In his book “Vehicles,” Braitenberg (1984) suggests replacing the analytical from-complex-to-simple perspective by a synthetic from-simple-to-complex approach. He introduces the latter by means of an extended thought experiment, in which he considers construing artificial creatures and contemplates about the psychological phenomena the behavior of these creatures would evoke in the naïve observer. Even surprisingly simple mechanisms, so he argues, may account for rather complex-looking phenomena. We do not advocate this particular empirical approach but find the theoretical attitude very promising. We therefore shall freely translate this attitude into an alternative research strategy that does not take phenomena but basic processes as a starting point.

As we have argued, basic processes (irrespective of the particular level or grain size at which they are defined) are likely to contribute to multiple subfunctions and phenomena. A synthetic approach could therefore start with a given basic process and investigate which aspects of which phenomena this process may account for. As an example, we and our colleagues have applied the rather simple idea that individuals may differ with respect to the degree of exclusivity to which the neural winner-takes-all principle is applied: some may strongly favor one representation over all competing representations, while others may only weakly favor one representation over its competitors. If so, this would predict systematic individual and group differences in performance profiles with respect to any phenomenon that is sensitive to this neural principle. Indeed, such differences were found with respect to phenomena as different as attentional control (Colzato et al., 2010), social cognition (Sellaro et al., 2014), creative thinking (Colzato et al., 2015a), meditation (Colzato et al., 2015b), and religious belief (Hommel et al., 2011)—e.g., religious beliefs favoring individualistic, exclusive thinking and decision-making lead to more attention to detail, better suppression of irrelevant information, less social integration, and greater patience than religious beliefs favoring collectivistic, inclusive processing.

Note that such a process-centered, synthetic approach does not provide an exhaustive model of the phenomena being considered (e.g., it would be hopeless to try explaining all aspects of religious belief by referring to a single neural principle). That is, it is far from accounting for all the variance of even a single to-be-explained phenomenon, but it does account for some variance of many different phenomena, as indicated in the right panel of Figure 1. In associating the same process with different phenomena, this approach also avoids identifying a process with a particular phenomenon, and the systematic search through different phenomena avoids phenomenon-specific specialization. It also comes with challenges, as it requires expertise in various experimental paradigms and theoretical frameworks, and publications in different journals with different customs. And yet, we are convinced that cognitive science would be well served by taking our historical lessons to heart and avoid repeating at least the most obstructive detours on the way to a deeper understanding of human cognition.

### Conflict of interest statement

The authors declare that the research was conducted in the absence of any commercial or financial relationships that could be construed as a potential conflict of interest.
